# Supplementary material for: Determination of Trace Lead and Cadmium in Decorative Material Using Disposable Screen-Printed Electrode Electrically Modified with Reduced Graphene Oxide/L-Cysteine/Bi-Film
Source: Sensors (Basel). 2020 Feb 28;20(5):1322. doi: 10.3390/s20051322 (PMC7085703; doi:10.3390/s20051322)
Supplement: Supplementary file 1 [file sensors-20-01322-s001.pdf]

Supporting Information

Determination of trace lead and cadmium in decorative material using disposable screen-printed electrode electrically modified with reduced graphene oxide/L-Cysteine/Bi-film

Xiaopeng Hou [1], Benhai Xiong [2], Yue Wang [2], Li Wang [3] and Hui Wang [1,2,*]

[1] Research Institute of Forestry New Technology and Research Institute of Wood Industry, Chinese Academy of Forestry, Beijing 100091, China; houxiaopeng_lunwen@163.com

[2] State Key Laboratory of Animal Nutrition, Institute of Animal Science, Chinese Academy of Agricultural Sciences, Beijing, 100193, China; xiongbenhai@caas.cn (B. H.) and wangyue9313@163.com (Y. W.)

[3] Geographic Information Center of Yulin City, Shannxi 719000, China. rnjwl612@163.com

* Correspondence: Hui Wang, Associate Research Assistant, Email: wanghui_lunwen@163.com and wanghuilunwen@cau.edu.cn

1. Optimization of Experimental Parameters

To further optimize the performance of Bi/LC-rGO/DSPE, some experimental parameters (pH, Bi(III) concentration, deposition time and deposition potential) were optimized in 0.1 mol/L acetate buffer solution containing 30 μg/L Cd(II) and Pb(II).

The acetic acid buffer solution can offer low background current, but the pH had an impact on the stripping response. In Figure S1, the stripping responses of Cd(II) and Pb(II) were changing with the pH in the range from 3.0 to 5.5. It was clear that the highest stripping responses of Cd(II) and Pb(II) were at pH 4.5. When pH was lower than 4.5, the stripping responses of Cd(II) and Pb(II) were weak. This phenomenon might be the hydrogen evolution reaction on the Bi/LC-rGO/DSPE in excessively acidic circumstances. However, the stripping responses of Cd(II) and Pb(II) were still low in the environment with high pH, which was mainly due to the hydrolysis reaction of bismuth that made it difficult to form bismuth-film effectively. Therefore, pH 4.5 was chosen for further study.

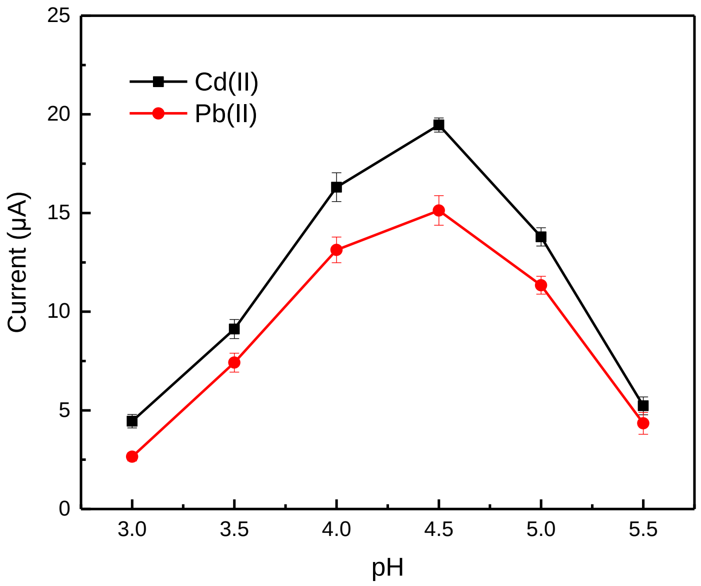

Figure S1. The stripping responses of Cd(II) and Pb(II) effected by the different pH of the acetic acid buffer solution.

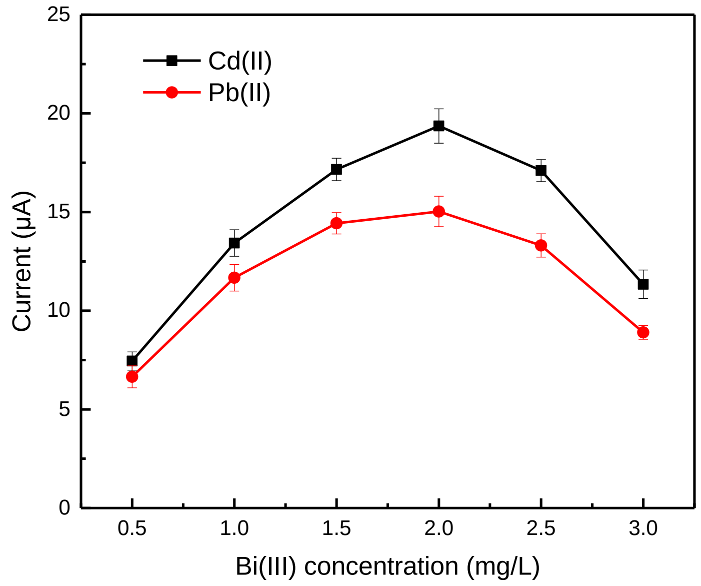

Figure S2. The stripping responses of Cd(II) and Pb(II) effected by the different Bi(III) concentrations.

The thickness of bismuth film on LC-rGO/DSPE had a severe impact on the stripping responses of Cd(II) and Pb(II). Figure S2 shows the relationship between different levels of Bi(III) and the stripping responses of 30.0 μg/L Cd(II) and Pb(II). The stripping responses increased quickly along with the increase of Bi(III) concentration from 0 to 2 mg/L, contributing that Cd(II) and Pb(II) were more comfortable to form

a binary alloy with bismuth. Further increasing the Bi(III) concentration, the sensitivity of Bi/LC-rGO/DSPE presented a decreasing trend. The reason was the bismuth film was too thick on the LC-rGO/DSPE surface that reduced the amount of dissolved Cd(II) and Pb(II). Therefore, the optimal Bi(III) concentration was selected as 2.0 mg/L.

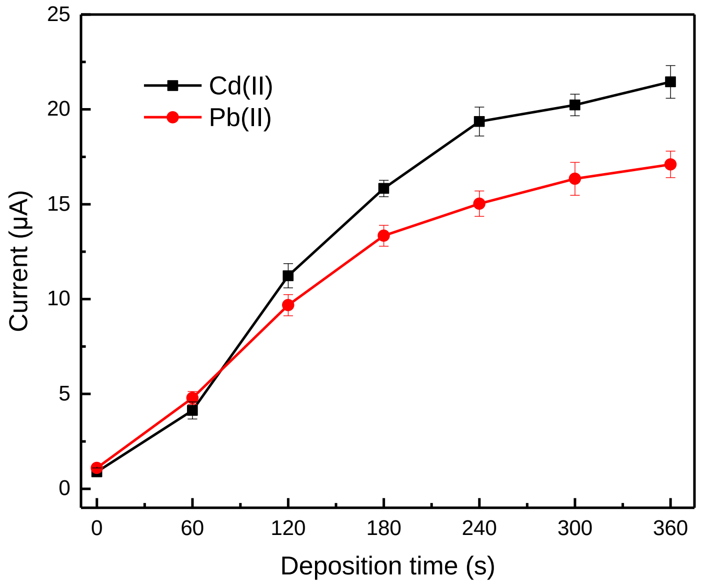

Figure S3. The stripping responses of Cd(II) and Pb(II) effected by the deposition times.

The deposition time had a significant influence on the stripping response. Figure S3 shows that the stripping responses of Cd(II) and Pb(II) were influenced by deposition time ranging from 60 to 360 s. When the deposition time was less than 240 s, the stripping responses increased significantly with the increase of deposition time. The stripping responses still increased to some extent while the deposition time beyond 240 s, but the growth rate appeared a decreasing tend because the deposited heavy metals become saturated on the Bi/LC-rGO/DSPE surface. Therefore, 240 s was chosen as the optimal deposition time.

The deposition potential affected the stripping responses of Cd(II) and Pb(II) was shown in Figure S4. The stripping responses of Cd(II) and Pb(II) were increasing significantly when the deposition potential was decreasing from −0.9 V to −1.2 V. At the deposition potential of −1.2 V, the highest stripping responses was achieved. With the deposition potential further negatively shifted more negative potential, the stripping responses decreased gradually, which was ascribed that the over negative deposition potential was easy to cause hydrogen evolution reaction. Thus, −1.2 V was chosen as the deposition potential.

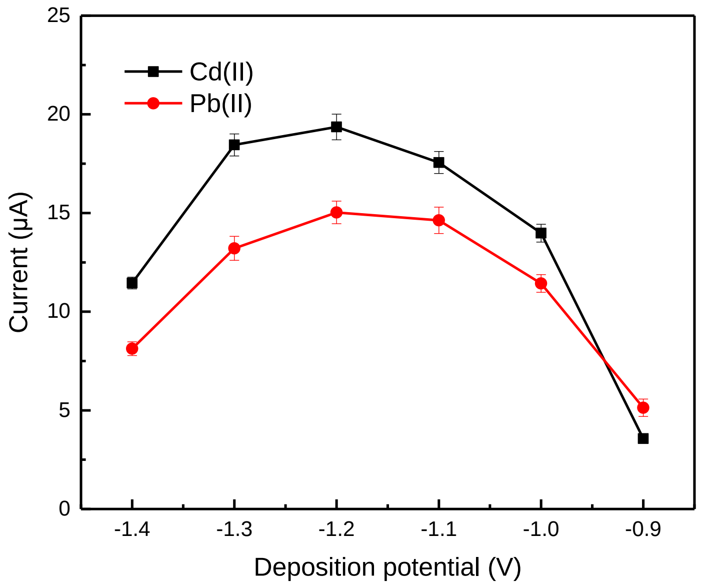

Figure S4. The stripping responses of Cd(II) and Pb(II) effected by the deposition potentials.

2. *The Stability*

To evaluate the stability, one electrode was selected to measure 20 μg/L Cd(II) and Pb(II) every two days under the same conditions. When not used, the electrode was stored in a natural environment at room temperature. In Figure S5, it was clear that the maximum deviation of the multiple measurements was less than 7.4%, indicating that the proposed electrode had good stability.

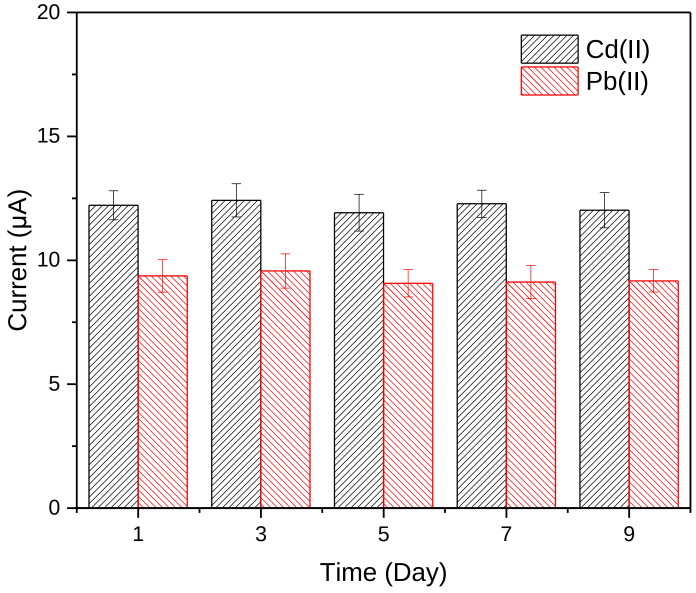

Figure S5. The stability of Bi/LC-rGO/DSPE measured 20 μg/L Cd(II) and Pb(II) every two days.
